# Impact of the neonatal intensive critical ultrasound workflow for managing neonatal acute pulmonary hypertension

**DOI:** 10.3389/fped.2026.1896889

**Published:** 2026-08-07

**Authors:** Mingsheng Zheng, Wenwei Ling, Jingyi Zhang, Rong Ju, Tiantian Xiao, Yiyong Fu, Biao Li, Yi Zheng, Jun Wang, Xiaofeng Zhou, Gaoyang Qin, Lingping Zhong, Ling Zhu, Youning Hu, Xiaolong Zhang, Zhengwei Ye, Huaying Li, Yang Liu, Nana Wu, Shuqiang Gao, Xuhong Hu, Yan Jiang, Xiaohong Luo

**Affiliations:** 1 Department of Neonatology, Chengdu Women's and Children's Central Hospital, School of Medicine, University of Electronic Science and Technology of China, Chengdu, China; 2 Department of Pediatrics, The Affiliated Hospital of Southwest Medical University, Luzhou, China

**Keywords:** acute pulmonary hypertension, aPH, critical ultrasound, neonate, NICUltra

## Abstract

**Objective:**

To investigate the value of developed the Neonatal Intensive Critical Ultrasound (NICUltra) Examination workflow in the management of neonatal acute pulmonary hypertension (aPH).

**Methods:**

In this retrospective cohort study, we assessed the effects of NICUltra-guided care on neonates with early aPH who underwent inhaled nitric oxide (iNO) therapy. The primary outcomes were the durations of iNO treatment and mechanical ventilation.

**Results:**

Of the 77 enrolled neonates, 36 (46.7%) received NICUltra-guided care. Compared with non-NICUltra-guided patients, NICUltra-guided patients had significantly shorter durations of iNO treatment (*P* = 0.002) and mechanical ventilation (*P* = 0.003) and lower hospitalization costs, whereas no between-group differences were detected in mortality, the incidence of severe intraventricular hemorrhage, or extracorporeal membrane oxygenation therapy utilization.

**Conclusion:**

Among neonates >32 weeks with early aPH receiving iNO treatment and without structural cardiopulmonary anomalies in a single-center retrospective study, the implementation of the NICUltra workflow is associated with significantly improved clinical efficacy and may be useful for optimizing clinical strategies with no observed differences in short-term outcomes.

## Highlights

What is Known:

1. Neonatal acute pulmonary hypertension (aPH) is one of the most challenging conditions to manage in the neonatal intensive care unit (NICU), which causes serious complications and even death.
2. Echocardiography is not only the “gold standard” for the diagnosis of aPH and phenotyping the aPH, but also it could optimize the management of aPH regarding the cardiorespiratory therapy and aPH utilization.

What is new:

1. In this study, we compared the clinical management and outcomes of neonates with aPH before and after the application of NICUltra workflow, and found that the iNO and mechanical ventilation duration of NICUltra-guided care group were significantly reduced, and the hospitalization cost was significantly reduced as well.
2. This suggests that the introduction of NICUltra workflows can improve clinical management of aPH.

## 1 Introduction

Neonatal acute pulmonary hypertension (aPH) is a heterogeneous disease that contributes significantly to as much as 32% of early mortality and 30%–35% of neurodevelopmental morbidity in this population (1–3). Most infants diagnosed with aPH present symptoms soon after birth, and their conditions are characterized primarily by refractory hypoxemic respiratory failure (HRF) with or without oxygen disparity. Persistent pulmonary hypertension of the newborn (PPHN) is a commonly used term to describe a clinical syndrome characterized by HRF secondary to an abnormal transition of the cardiopulmonary circulation in the immediate postnatal period (4). The incidence of aPH has been reported to be approximately 0.43–6 in 1,000 live term infants (5). In China, accurate epidemiological data on aPH are lacking; however, approximately 3.4% of neonates with HRF require inhaled nitric oxide (iNO) postnatally (6). Notably, aPH places a significant burden on healthcare systems and families because of prolonged hospitalization, specialized care, and potential long-term effects (7, 8).

The gold standard for diagnosing aPH is based on cardiac catheterization in adults; however, its application in neonates is challenging (9). Therefore, in practice, the diagnosis of aPH is mainly based on clinical features and echocardiography data. The determinants of neonatal pulmonary hypertension are pulmonary vascular resistance (PVR), pulmonary blood flow (PBF), and pulmonary capillary wedge pressure (PCWP) (9). Furthermore, neonates with aPH could be complicated with cardiac dysfunction secondary to hypoxia (10). The sixth World Symposium on Pulmonary Arterial Hypertension guidelines recommend the classification of neonatal aPH according to etiology (11). Previous data revealed that 80%–90% of aPH cases were caused by infection (30%), meconium aspiration syndrome (24%), and respiratory distress syndrome (7%) (5), etc. Accordingly, the etiology, underlying determinants of aPH, and cardiac performance are the main considerations for managing aPH in neonates. However, identifying the above critical information solely based on the clinical history and presentations in practice is difficult. Therefore, implementing a comprehensive clinical assessment combined with emerging echocardiography measures to evaluate the major contributors to aPH and myocardial performance has been suggested to be a more useful approach for the management of aPH and could provide critical decision-making support for clinicians to administer precision therapies and help decrease morbidity and mortality (12–14).

Unlike targeted neonatal echocardiography (TNE), which focuses on cardiac function, the Neonatal Intensive Critical Ultrasound (NICUltra) Examination workflow (15), which was developed by our team and has been in use since June 2022, integrates pulmonary function, cardiac function, and end-organ blood flow to guide the diagnosis and management of respiratory failure and hemodynamic instability in critically ill neonates. In this study, we conducted a retrospective analysis of clinical data from neonates with aPH at our center to evaluate the effects on clinical strategies and outcomes before and after the implementation of the NICUltra workflow.

## 2 Materials and methods

### 2.1 Study design and population

This retrospective, single-center, before-and-after cohort study was performed at a level IV NICU in Chengdu Women and Children's Central Hospital in China. This study was approved by the Institutional Research Ethics Board [2022(14)].

We evaluated all neonates with aPH who received iNO treatment between January 1, 2021, and May 31, 2022, and between June 1, 2022, and December 31, 2023. The cardiovascular care policy has changed since June 1, 2022, in our NICU. Since then, we have implemented the NICUltra workflow for the management of critically ill neonates as a protocol (15). Therefore, the NICUltra-guided care aPH group was enrolled from a registered cohort (ChiCTR2200065581). Neonates with aPH who received iNO between January 1, 2021, and May 31, 2022, composed our historical cohort.

Our internal healthy cohort was designated as the control group at a 1:2 ratio based on GA, BW, sex, and the time at which NICUltra was performed during our new cardiovascular care policy period to further analyze the hemodynamic characteristics of neonates with aPH. The internal healthy cohort was selected from the cohort for the establishment of the ultrasonic reference range in healthy newborns, which has been registered in the Chinese Clinical Trial Registry (ChiCTR2300074563).

The inclusion criteria were as follows: (1) neonates who were diagnosed with aPH and treated with iNO and (2) biological parents or guardians who consented to participate in this study (the parental consent waiver granted for the historical cohort between January 1, 2021, and May 31, 2022). Neonates were excluded if (1) their gestational age (GA) was < 32 weeks; (2) their birth weight (BW) was < 1,500 g; (3) they had cyanotic critical congenital heart disease; (3) their aPH was related to genetic disorders or congenital pulmonary dysplasia (such as congenital diaphragmatic hernia); or (4) their parents wanted palliative care before the end of iNO treatment. The clinical diagnosis and management were reviewed by two senior neonatologists. The diagnosis of aPH was primarily based on clinical criteria with or without echocardiography and was consistent across all the enrolled neonates (details are provided in Supplementary Material respectively).

### 2.2 Statistical analysis

The data were analyzed using R v4.4.3 (The R Core Team, 2024; The R Foundation for Statistical Computing, Vienna, Austria). Normally distributed measurement data are presented as the means ± standard deviations (X ± s), and two independent samples t tests were used for comparisons between two groups. Nonnormally distributed measurement data are presented as medians with interquartile ranges (IQRs), and the Mann–Whitney U test was used for comparisons between two groups; enumeration data are presented as numbers and percentages. Comparisons between groups were performed using the chi-square test or Fisher's exact test. *P* < 0.05 was considered to indicate statistical significance.

## 3 Results

### 3.1 Study cohort

**Figure 1:**
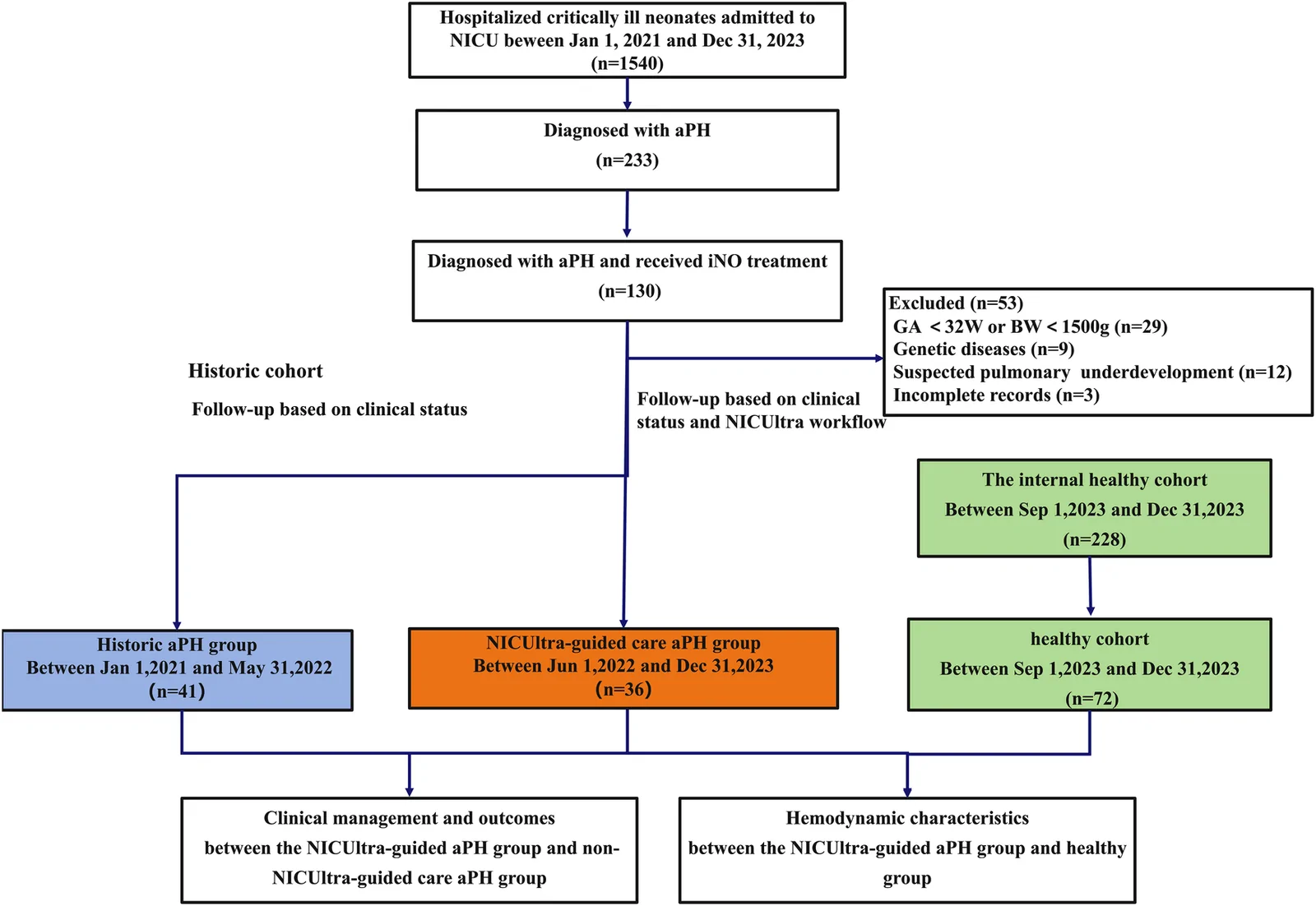
Flow chart of enrolled subjects. NICU, neonatal intensive care units; aPH, acute pulmonary hypertension; iNO, inhaled nitric oxide; GA, gestational age; BW, birth weight; NICUltra, neonatal intensive critical ultrasound.

Among the 1,540 critically ill neonates, 233 were diagnosed with aPH, and 77 were included in the study cohort (Figure 1). Among these neonates, 46 were male and 31 were female, with a mean GA of 37.5 (2.1) weeks and a mean BW of 3,036.2 (546.1) grams. All patients survived and did not require ECMO. The median age of initiation of iNO treatment was 23.0 (11.0, 29.0) hours. A total of 36 (46.7%, 36/77) neonates required NICUltra-guided care (Table 1 and Supplementary Table S1).

**Table 1:** The characteristics of neonates with acute pulmonary hypertension.

Characteristics	Total (*n* = 77)	NICUltra-guided care aPH group (*n* = 36)	Historic aPH controls (*n* = 41)	P-value
Perinatal conditions				
Male, *n* (%)	46 (59.7)	22 (61.1)	24 (58.5)	0.818
GA (weeks), mean (SD)	37.4 (2.1)	37.5 (2.2)	37.3 (2.0)	0.594
BW (grams), mean (SD)	3,022.05 (545.9)	3,070.14 (565.5)	2,978.41 (554.6)	0.475
Outborn, *n* (%)	45 (58.4)	23 (63.9)	22 (53.7)	0.487
Cesarean Section, *n* (%)	54 (70.1)	24 (66.7)	30 (73.2)	0.534
GDM, *n* (%)	11 (14.3)	5 (13.9)	6 (14.6)	0.926
Chorioamnionitis, *n* (%)	2 (2.6)	1 (2.8)	1 (2.4)	1.000^a^
ICP, *n* (%)	12 (15.6)	5 (13.9)	7 (17.1)	0.701
PIH, *n* (%)	6 (7.8)	2 (5.6)	4 (9.8)	0.679^a^
OI, M (Q1, Q3)	30 (11.2, 27.0)	17.30 (11.2, 23.8)	17.8 (11.5, 28.4)	0.907
SNAPPE2 score, M (Q1, Q3)	21 (10, 22)	21 (10, 21)	21 (10, 25)	0.796
Age at iNO (h), M (Q1, Q3)	23.0 (11.0, 29.0)	25 (13, 31)	20.0 (8.5, 27.5)	0.279
aPH etiology				
RDS, *n* (%)	57 (74.0)	23 (63.9)	34 (82.9)	0.057
MAS, *n* (%)	12 (15.6)	7 (19.4)	5 (12.2)	0.382
ALS, *n* (%)	23 (29.9)	11 (30.6)	12 (29.3)	0.902
Asphyxia, *n* (%)	19 (24.7)	9 (25.0)	10 (24.4)	0.951
EOS, *n* (%)	54 (70.1)	22 (61.1)	32 (78.0)	0.105

aPH, acute pulmonary hypertension; NICUItra, neonatal intensive critical ultrasound; GA, gestational age; BW, birth weight; GDM, gestational diabetes mellitus; ICP, intrahepatic cholestasis of pregnancy; PIH, pregnancy-induced hypertension; OI, oxygen index (The peak value occurred within 72 h post admission); SNAPPE2, Score for neonatal acute physiology, perinatal extension, version II (The peak value occurred within 72 h post admission); iNO, inhale nitric oxide; RDS, respiratory distress syndrome; MAS, meconium aspiration syndrome; ALS, air leak syndrome; EOS, early-onset sepsis.

a fisher's exact test.

### 3.2 Hemodynamic characteristics in the NICUltra-guided care aPH group and healthy group

With respect to cardiac function, a lower TAPSE (6.90 mm vs. 8.50 mm, *p* < 0.001), higher TVE (61.70 cm/s vs. 49.75 cm/s, *p* = 0.010), higher TV E/A ratio (1.00 vs. 0.81, *p* = 0.001), lower left ventricular end-systolic diameter (LVID) (12.00 mm vs. 18.30 cm, *p* < 0.001), lower MAPSE (5.25 mm vs. 6.30 mm, *p* < 0.001), and lower left ventricular CO (102.74 mL/kg vs. 132.46 mL/kg, *p* < 0.001) were detected in neonates with aPH. The LUS was high [22 (20, 26)] in the aPH group. Regarding the MCA, compared with the healthy group, the neonates with aPH presented a higher peak systolic velocity (PSV) (left: 58.9 cm/s vs. 45.80 cm/s, *p* = 0.028; right: 63.90 cm/s vs. 47.20 cm/s, *p* < 0.001) (Supplementary Table S2).

### 3.3 Clinical management of the NICUltra-guided aPH group and the historic aPH controls

Statistical analysis revealed that the durations of iNO treatment [62.00 [37.50–73.00] hours vs. 83.00 [63.00–111.00] hours, *P* = 0.002] and invasive mechanical ventilation [155.50 [127.00–181.25] hours vs. 184.00 [162.00–230] hours, *P* = 0.003] were significantly shorter in the NICUltra-guided care aPH group than in the Historic aPH controls. A significant difference in the failure to wean off invasive mechanical ventilation was not observed (2.9% vs. 4.9%, *P* = 1.000). Compared with the Historic aPH controls, the NICUltra-guided care aPH group showed a significant changes in dopamine (66.7% vs. 95.1%, *P* = 0.001) and milrinone usage (52.8% vs. 17.1%, *P* < 0.001) (Table 2).

**Table 2:** The outcome of the neonates with acute pulmonary hypertension between the NICUltra-guide care aPH group and historic aPH controls.

Outcome	Total (*n* = 77)	NICUltra-guided care aPH group (*n* = 36)	Historic aPH controls (*n* = 41)	Difference (95%CI)	P-value
Primary outcome					
iNO duration (h), M (P25-P75)	69 (50–92)	62 (37.5–73)	83 (63–111)	-21 (-37 to -4.49)	0.002
MV duration (h), M (P25-P75)	168 (136–208)	155.5 (127–181.25)	184 (162–230)	-28.5 (-59.51 to -9)	0.003
Secondary outcome					
PS usage, M (P25-P75)	1 (0–1)	1 (0–1)	1 (1–1)	0 (-1 to 0)	0.035
Failure of wean off the invasive mechanical ventilation, *n* (%)	3 (3.9)	1 (2.8)	2 (4.9)	-2.1 (-10.6 to 6.4)	1.000^a^
VIS, M (P25-P75)	19 (8–29)	21 (12–30)	16 (7–28)	5 (-4 to 13.5)	0.295
Dopamine, *n* (%)	63 (81.8)	24 (66.7)	39 (95.1)	-28.5 (-45.2 to -11.7)	0.001
Dobutamine, *n* (%)	34 (44.2)	12 (33.3)	22 (53.7)	-20.3 (-42.0 to 1.3)	0.073
Milrinone, *n* (%)	26 (33.8)	19 (52.8)	7 (17.1)	35.7 (15.7 to 55.7)	<0.001
Norepinephrine, *n* (%)	47 (61.0)	25 (69.4)	22 (53.7)	15.8 (-5.6 to 37.2)	0.156
Epinephrine, *n* (%)	22 (28.6)	14 (38.9)	8 (19.5)	19.4 (-0.6 to 39.4)	0.060
Vasopressin, *n* (%)	0 (0)	0 (0)	0 (0)	0 (-4.8 to 5.1)	1.000^a^
Number of vasoactive agents used					
Single agent, *n* (%)	3 (3.9)	0 (0)	3 (7.3)	-7.3 (-16.1 to 2.2)	0.243^a^
Two combination agents, *n* (%)	35 (45.5)	17 (47.2)	18 (43.9)	3.3 (-18.9 to 25.6)	0.770
Three combination agents, *n* (%)	29 (37.7)	14 (38.9)	15 (36.6)	2.3 (-19.4 to 24.0)	0.835
> 3 combination agents, *n* (%)	7 (9.1)	4 (11.1)	3 (7.3)	3.79 (-9.2 to 16.8)	0.699
Mortality at discharge, *n* (%)	0 (0)	0 (0)	0 (0)	0 (-4.8 to 5.1)	1.000^a^
Required ECMO, *n* (%)	0 (0)	0 (0)	0 (0)	0 (-4.8 to 5.1)	1.000^a^
Severe IVH, *n* (%)	5 (6.5)	2 (5.6)	3 (7.3)	-1.7 (-12.7 to 9.2)	1.000^a^
Length of stay (d), M (P25-P75)	17 (15–21)	16 (14.5–20)	18 (15–23)	-2 (-4 to 2)	0.302
Cost ratio, M (P25-P75)	0.85 (0.67–1.03)	0.76 (0.63–0.91)	1.00 (0.74–1.13)	-0.24 (-0.32 to -0.05)	0.002

NICUItra, neonatal intensive critical ultrasound; aPH, acute pulmonary hypertension; iNO, inhaled nitric oxide; MV, mechanical ventilation; PS, pulmonary surfactant; VIS: vasoactive inotropic score; ECMO, extracorporeal membrane oxygenation; IVH, intraventricular hemorrhage. Cost ratio, the median cost of hospitalization in the Historic aPH controls is recorded as 1.00.

a fisher's exact test.

### 3.4 Clinical outcomes between the NICUltra-guided care aPH group and historic aPH controls

With respect to the outcomes at discharge, a lower cost ratio [0.76 [0.63–0.91] vs. 1.00 [0.74–1.13], *p* = 0.002] of hospitalization was observed in the NICUltra-guided care aPH group. No significant differences in mortality, the rate of severe IVH, the use of ECMO therapy or the length of stay were observed between these two groups (Table 2).

### 3.5 Sensitivity analysis of the primary outcome between the NICUltra-guide care aPH group and historic aPH controls

Table 1 indicates that the incidence of RDS was imbalanced between the two groups; therefore, a sensitivity analysis was performed. The results showed that in the RDS subgroup, the duration of iNO and mechanical ventilation in the NICUltra-guide care aPH group remained significantly shorter than that in the historic aPH group. In the no RDS subgroup, compared with the historic aPH group, the NICUltra-guided care aPH group demonstrated a downward trend in the duration of iNO and mechanical ventilation, although these differences did not reach statistical significance (*P* > 0.05) (Table 3).

**Table 3:** Sensitivity analysis of the primary outcome of the neonates with acute pulmonary hypertension between the NICUltra-guide care aPH group and historic aPH controls.

Primary outcome	Total (*n* = 77)	NICUltra-guided care aPH group (*n* = 36)	Historic aPH controls (*n* = 41)	Difference (95%CI)	P-value
iNO duration (h), M (P25-P75)					
RDS	73 (57–106)	66 (49–73)	89 (68–114)	-23 (-41 to -9)	0.006
No RDS	59 (33–74)	56 (33–72)	63 (50.5–74.5)	-9 (-29.5 to 11.5)	0.402
MV duration (h), M (P25-P75)					
RDS	180 (148–208)	160 (133.5–187.5)	186.5 (166–235.5)	-26.5 (-56.5 to -1.5)	0.010
No RDS	145.5 (117.5–185.5)	145 (117–176)	162 (128.5–216.5)	-23 (-68 to 22)	0.334

NICUItra, neonatal intensive critical ultrasound; aPH, acute pulmonary hypertension; iNO, inhaled nitric oxide; RDS, respiratory distress syndrome; MV, mechanical ventilation.

## 4 Discussion

This study is the first to perform an integrated assessment of cardiac function, pulmonary function and end-organ blood flow in neonates with aPH. In this study, the NICUltra-guided care aPH group exhibited a significantly shorter duration of iNO treatment and mechanical ventilation than did the Historic aPH controls. The majority of treatment costs for neonates with aPH are attributed to the duration of iNO treatment and mechanical ventilation; therefore, reducing the durations of these treatments can effectively lower treatment expenses.

Multiple potential etiologies of aPH exist, including MAS, infection, RDS, HIE, and pulmonary hemorrhage. Physiologically, aPH can occur from high PVR, high PBF and elevated PCWP (10, 16). In our group, high PVR was the only identified contributor to aPH, which was secondary to RDS (74%) and sepsis (70%). Furthermore, recent targeted neonatal echocardiography (TNE) studies of aPH secondary to high PVR have suggested that four phenotypes should generally be considered before initiating treatment (iNO therapy or inotropes): a high PVR without compromised biventricular function, high PVR with predominant right ventricular dysfunction, high PVR with predominant left ventricular dysfunction, and high PVR with predominant biventricular dysfunction. In practice, sick neonates can present with HRF and shock; however, routine clinical exams cannot be used to detect these conditions. Consistent with these studies (10, 16), the echocardiographic markers among our hemodynamic data in this cohort, such as lower cardiac output, MAPSE, MVE, TAPSE, and TVE and a higher TV E/A ratio, indicated that neonates with aPH could present left, right, or biventricular dysfunction.

These different phenotypes emphasize the importance of integrating echocardiographic assessments to tailor pulmonary vasodilators, inotropes, and vasoconstrictors, or PGE. Studies have reported that the duration of iNO treatment varies from 1 to 31 days (17, 18), and our duration of iNO treatment was significantly reduced by approximately 30% (from 83.00 h to 62.00 h) after the implementation of the NICUltra workflow. Consistent with the study by Joye et al. (13), we found that the proportions of patients treated with inotropes or vasopressors changed when ultrasonic information was provided to neonatologists. Milrinone and epinephrine were used more frequently to improve cardiac function in the NICUltra-guided care group than in the non-NICUltra-guided care aPH group, which is consistent with the echocardiographic findings in our study.

aPH may further decrease cerebral oxygenation, impact cerebral hemodynamics, and cause additional brain injury in addition to the potential etiologies (19, 20). Approximately 30%–35% of survivors with aPH continue to have one or more neurodevelopmental disabilities (21, 22). Furthermore, an animal study revealed the potential mechanism underlying the involvement of the brain in PH pathophysiology (23). In our study, we analyzed the blood velocity of the middle cerebral artery (MCA), which can serve as a surrogate hemodynamic marker for assessing cerebral circulation. We found that the peak systolic velocities in both the right and left MCAs were greater than those in the control group. This result might be an adaptation to maintain adequate cerebral oxygenation by increasing blood flow. A previous animal study revealed increased cerebral flow in an aPH model (24). However, cerebral circulation is regulated by several factors, such as metabolic acidosis and CO2, and autoregulation changes at different developmental stages during the postnatal period. Therefore, these findings should be interpreted cautiously. The associations between cerebral circulation and PH need to be further investigated.

With respect to the management of aPH, lung optimization is always critical. Lung ultrasound has been proven to be an important bedside assessment tool in critical care (25). Therefore, in our NICUltra workflow, we integrated lung and cardiac assessments in this cohort. As shown in the study by Singh et al. (26), lung ultrasound can aid in the diagnosis of RDS, pneumothorax, and MAS. Additionally, LUS scores can be used to assess the lung fluid content. A systematic review suggested that an LUS score greater than 8 can indicate the need for surfactant (27). Our initial median LUS score was 22, suggesting that parenchymal lung diseases were severe in our cohort. Furthermore, the prolonged use of invasive mechanical ventilation increases the risk of neonatal lung injury, neurodevelopmental disorders, and ventilator-associated pneumonia (28, 29). An LUS assessment is one part of our workflow. LUS can be used to effectively identify whether mechanically ventilated infants are ready for weaning or require increased respiratory support or positional therapy (30). Studies have shown that LUS can be used to detect pulmonary edema and pulmonary consolidation in neonates, even when chest x-rays do not reveal any signs of these conditions, allowing targeted interventions that promote successful extubation (31–33). Our study revealed that the the duration of invasive ventilation in the NICUltra-guided care aPH group was 1 day shorter than that in the historic aPH controls. However, no significant difference in the median hospital stay was observed between the two groups. A detailed analysis revealed that the factors delaying discharge for neonates remaining hospitalized in the NICUltra-guided group could be attributed to the following clinical situation: completion of antibiotic courses (18/34, 52.9%), hospital-acquired infections (2/34, 5.9%), and difficult oral feeding (6/34, 17.6%).

The current study has several limitations. First, our study was conducted retrospectively at a single center, which may introduce inherent selection bias and a small sample size. The mortality and ECMO utilization in both groups were zero, and severe IVH was extremely rare. The sample size of this study was insufficient to detect clinically important differences in these outcomes, limiting the reliability of safety inferences. Second, with respect to the hemodynamic measures, some measures associated with right heart function, such as the right ventricular fractional area of change and right ventricular output, were not investigated (34). We used the PDA flow direction, TR jet velocity, and qualitative septum configuration to assess pulmonary artery pressure in our initial NICUltra protocols. In accordance with recent recommendations from the 2024 American Society of Echocardiography guidelines (35), more echocardiographic markers of a high PVR, such as right ventricular ejection time (RVET)/pulmonary artery acceleration time (PAAT) and the LV-end-systolic eccentricity index (EI), are available. Further studies could validate these echocardiographic markers for aPH assessment. The NICUltra workflow is our initial version, and this version will be revised further. Third, the study spanned a period of three years, and inflation was not taken into account. The hemodynamic characteristics might not be typical in this cohort.

In conclusion, Among neonates >32 weeks with early aPH receiving iNO treatment and without structural cardiopulmonary anomalies in a single-center retrospective before-after study, the implementation of the NICUltra workflow for guiding the clinical management of aPH can significantly reduce the duration of iNO treatment and mechanical ventilation without observed differences in adverse outcomes, thereby presenting certain economic advantages, but prospective multicenter validation is needed.

## Data Availability

The raw data supporting the conclusions of this article will be made available by the authors, without undue reservation.
